# Differential Physiological Responses to Salt Stress between Salt-Sensitive and Salt-Tolerant *japonica* Rice Cultivars at the Post-Germination and Seedling Stages

**DOI:** 10.3390/plants10112433

**Published:** 2021-11-11

**Authors:** Shenghai Ye, Zhibo Huang, Guibin Zhao, Rongrong Zhai, Jing Ye, Mingming Wu, Faming Yu, Guofu Zhu, Xiaoming Zhang

**Affiliations:** 1 Institute of Crop and Nuclear Technology Utilization, Zhejiang Academy of Agricultural Sciences, Hangzhou 310021, China; shenghaiye@163.com (S.Y.); zhxr729@163.com (R.Z.); yejingzj@163.com (J.Y.); wumm@zaas.ac.cn (M.W.); yfm4679@163.com (F.Y.); zhuguofu65@126.com (G.Z.); 2 The Laboratory of Seed Science and Technology, Guangdong Key Laboratory of Plant Molecular Breeding, Guangdong Laboratory of Lingnan Modern Agriculture, State Key Laboratory for Conservation and Utilization of Subtropical Agro-Bioresources, South China Agricultural University, Guangzhou 510642, China; 20202015013@stu.scau.edu.cn (Z.H.); zhaoguib23@163.com (G.Z.)

**Keywords:** *Oryza sativa*, salt stress, Na^+^/K^+^ homeostasis, hydrogen peroxide, seed germination, seedling growth

## Abstract

Soil salinity is a key source of abiotic stress in the cultivation of rice. In this study, two currently cultivated *japonica* rice species—Zhegeng 78 (salt-tolerant) and Zhegeng 99 (salt-sensitive)—with similar backgrounds were identified and used to investigate their differential responses to salt stress at the post-germination and seedling stages. Quantitative RT-PCR analysis demonstrated that the expression of *OsSOS1*, *OsHAK1*, and *OsHAK5* at the post-germination stage, and the expression of *OsHKT1,1*, *OsHTK2,1*, and *OsHAK1* at the seedling stage, were significantly higher in the salt-tolerant Zhegeng 78 compared with those of the salt-sensitive Zhegeng 99 under salt stress. The significantly lower Na^+^ net uptake rate at the post-germination and higher K^+^ net uptake rates at the post-germination and seedling stages were observed in the salt-tolerant Zhegeng 78 compared with those of the salt-sensitive Zhegeng 99 under salt stress. Significantly higher activity of peroxidase (POD) and the lower hydrogen peroxide (H_2_O_2_) accumulation were observed in the salt-tolerant Zhegeng 78 compared with those of salt-sensitive Zhegeng 99 under salt stress at the seeding stage. The salt-tolerant Zhegeng 78 might be valuable in future cultivation in salinity soils.

## 1. Introduction

Rice (*Oryza sativa* L.) is a salt-sensitive crop [1,2]. It is estimated that approximately 30% of the world’s rice cultivation area is affected by salt stress [3]. Soil salinity is seriously influencing rice production, especially at the seed germination and seedling stages, due to the popularity of direct-seeding cultivation [4]. The improvement of seedling establishment under salt stress is critical for rice [2]. Therefore, the identification of salt-tolerant cultivars is one of the most important objectives of rice production in coastal areas. Illumination of the physiological responses to salt stress during the seed germination, post-germination and seedling stages would help us to select salt-tolerant varieties for rice cultivation in salinity soils.

The homeostasis of Na^+^/K^+^ ratio plays important roles in salt tolerance in plants. To date, numerous salt-responsive genes associated with Na^+^/K^+^ homeostasis have been identified in rice. For example, Salt Overly Sensitive 1 (SOS1), encoding the plasma membrane Na^+^/H^+^ exchanger protein, is the sole Na^+^ efflux transporter that has been characterized to control net root Na^+^ uptake and Na^+^ transport to shoots in rice [5]. The rice sodium transporter gene *OsHKT1;1* regulates both Na^+^ unloading from the xylem and Na^+^ loading into the phloem in leaves for Na^+^ recirculation [6]. Rice *OsHKT2;1* as a key sodium transporter affects Na^+^ uptake and the efficiency of K^+^ use [7,8]. Similarly, rice *OsHKT1;5* is also involved in the regulation of Na^+^/K^+^ ratio under salt stress [9]. Additionally, several high-affinity K^+^ transporters (HAKs), such as *OsHAK1* [10], *OsHAK5* [11], and *OsHAK21* [12,13], through the activation of the K^+^ uptake function, are also involved in the maintaining of the Na^+^/K^+^ ratio under salt stress in rice.

Reactive oxygen species (ROS) are small molecules that play a dual role in seed germination. The low level of ROS as signaling messengers leads to seed germination, whereas excessive accumulation of ROS inhibits germination [14]. The accumulation of ROS induced by salt stress will disturb ionic homeostasis in the cells, which will damage the key cellular structures. Therefore, it is important to keep the balance of ROS accumulation for successful seed germination and seedling growth under salt stress in rice. In plants, there are enzymic and non-enzymatic mechanisms for ROS scavenging [15]. For example, the enzymatic components, such as superoxide dismutase (SOD), peroxidase (POD), catalase (CAT), ascorbate peroxidase (APX), and glutathione reductase (GR), are involved in the regulation of ROS levels [16]. The associations between seed germination and the activities of SOD, CAT, AP, and GR were reported in rice [17]. Recently, it was reported that the enhancing of ROS detoxification by rice SAPK1 and SAPK2 mainly occurs through the increasing of SOD and CAT expression under salt stress [18]. 

Numerous studies have investigated salt tolerance using the wild rice accessions or landraces [13,19,20,21,22]; however, the characteristics of salt responses in the currently cultivated *japonica* rice have not been thoroughly investigated. In this study, in order to select the salt-tolerant *japonica* rice for direct seeding, the salt tolerance of six currently cultivated *japonica* varieties in Zhejiang Province (E 118°01′–123°10′, N 27°02′–31°11′) of China were firstly investigated at the post-germination and seedling stages. Two *japonica* cultivars, one salt-sensitive (Zhegeng 78) and one salt-tolerant (Zhegeng 99), with similar genetic backgrounds, were identified. It was interesting to identify the mechanism of differential salt tolerance among cultivars with similar genetic backgrounds. Then, the Zhegeng 78 and Zhegeng 99 were used to analyze their differential physiological responses to salt stress. The expression of genes associated with Na^+^/K^+^ homeostasis, the H_2_O_2_ level, and the POD and SOD activities were compared between the salt-sensitive Zhegeng 78 and salt-tolerant Zhegeng 99 cultivars. It was found that the identified salt-tolerant *japonica* cultivar Zhegeng 78 might be useful for the future cultivation of salinity soils.

## 2. Results

### 2.1. Phenotype of Salt Tolerance in Japonica Rice Cultivars

**Figure 1:**
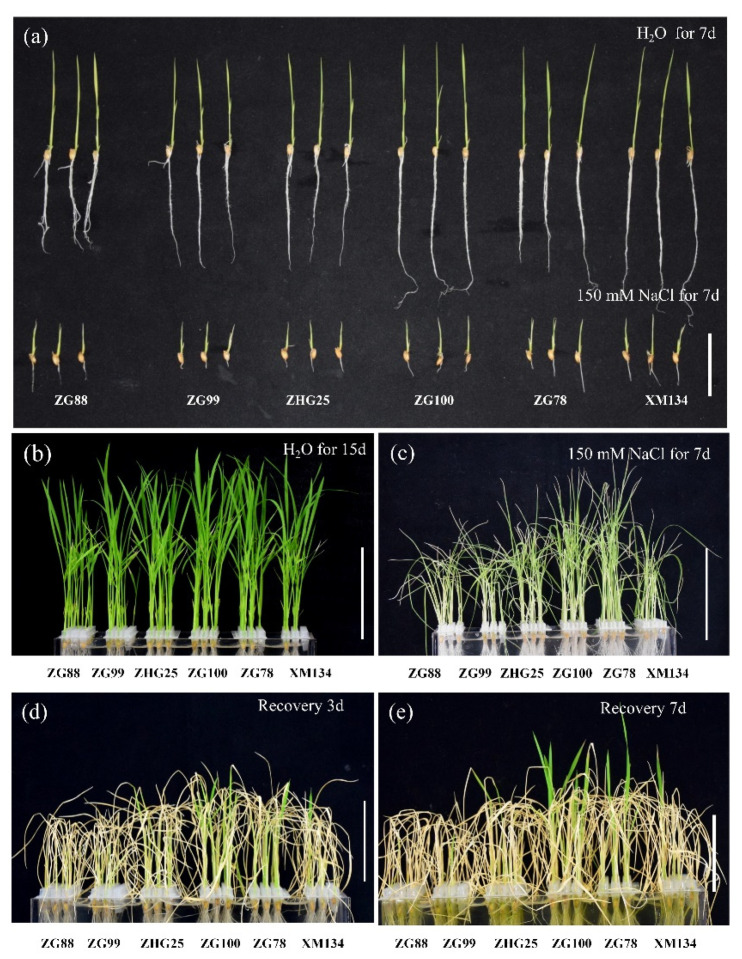
Phenotype of salt tolerance in *japonica* rice cultivars. (**a**) The early growth of the post-germinated seeds under H_2_O and 150 mM NaCl conditions for 7 days. (**b**) The seedlings’ growth at the three-leaf stage under H_2_O condition for 15 days. (**c**) The seedlings’ growth at the three-leaf stage treated after 150 mM NaCl for 7 days. The seedlings’ growth at the three-leaf stage treated after 150 mM NaCl for 7 days and with the recovery growth for 3 days (**d**) and 7 days (**e**). Bars = 5 cm.

In order to reveal the characteristics of salt tolerance in the currently cultivated *japonica* rice, six cultivars including Zhegeng 78 (ZG78), Zhegeng 88 (ZG88), Zhegeng 99 (ZG99), Zhegeng 100 (ZG100), Zhehugeng 25 (ZHG25), and Xiushui 134 (XS134), in Zhejiang Province of China, were used to evaluate the performance of seedling growth under salt stress (150 mM NaCl) at the post-germination and seedling stages. The post-germinated seeds (radicle length 5 mm) were treated under 150 mM NaCl for 7 days (Figure 1a). Similar shoot lengths were observed among cultivars and longer root lengths were observed in ZG100 and XS134 compared with those of other cultivars under normal (H_2_O) conditions. The influence of root length in six cultivars was more affected by salt stress than that of shoot growth. The root lengths of ZG78, ZG88, and XS134 were longer than those of ZG99, ZG100, and ZHG25 after salt treatment. Meanwhile, the seedlings of six cultivars at three-leaf stage were treated under 150 mM NaCl for 7 days and recovery growth for 7 days (Figure 1b–e). The seedling survival was higher in ZG78, ZG100, and XS134 than in ZG88, ZG99, and ZHG25 (Figure 1d,e). By comparison, ZG78 and XS134 had better salt tolerance simultaneously at both the post-germination and seedling stages, while ZG99 and ZHG25 had lower salt tolerance. 

### 2.2. Agronomic Traits and Stress Tolerance of Zhegeng 78 and Zhegeng 99

**Figure 2:**
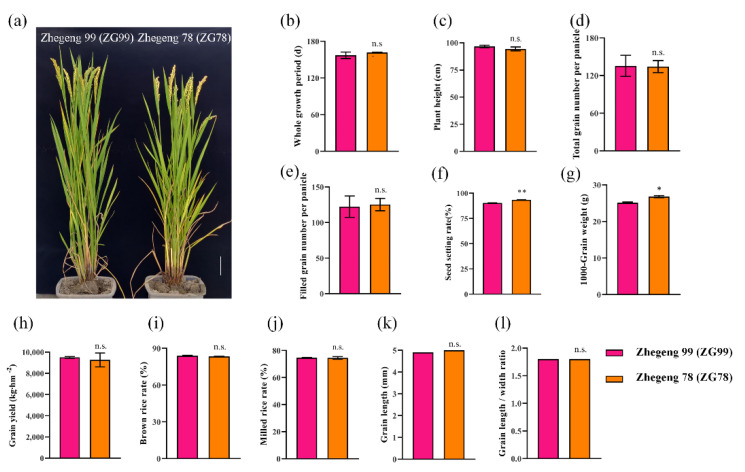
Comparison of agronomic traits between *japonica* rice cultivars Zhegeng 78 and Zhegeng 99. (**a**) Images of Zhegeng 99 and Zhegeng 78, Bar = 10 cm; (**b**) whole growth period; (**c**) plant height; (**d**) total grain number per panicle; (**e**) filled grain number per panicle; (**f**) seed setting rate; (**g**) 1000-grain weight; (**h**) grain yield; (**i**) brown rice rate; (**j**) milled rice rate; (**k**) grain length; (**l**) grain length/width ratio. Data are means (±SD), *n* = 2. Significant differences between cultivars were determined using Student’s *t*-test: * *p* < 0.05; ** *p* < 0.01. n.s. not significant.

**Figure 3:**
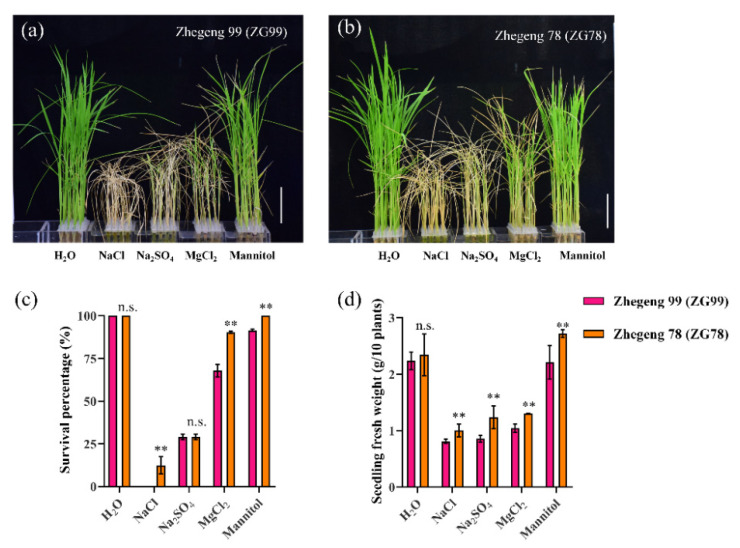
Comparison of stress tolerance between *japonica* rice cultivars Zhegeng 78 and Zhegeng 99. (**a**,**b**) The seedling growth of Zhegeng 99 and Zhegeng 78 at the three-leaf stage treated after H_2_O, 150 mM NaCl, 75 mM Na_2_SO_4_, 75 mM MgCl_2_, and 150 mM Mannitol for 7 days. Bars = 5 cm. (**c**) Survival percentage. (**d**) Seedling fresh weight. Data are means (±SD), *n* = 3. Significant differences between cultivars were determined using Student’s *t*-test: ** *p* < 0.01; n.s. not significant.

As mentioned above, Zhegeng 78 (ZG78) had higher salt tolerance at both the post-germination and seedling stages, while Zhegeng 99 (ZG99) had lower salt tolerance. Interestingly, ZG78 and ZG99 have similar genetic backgrounds. Similar traits for the whole growth period, plant height, total grain number per panicle, filled grain number per panicle, grain yield, brown rice rate, milled rice rate, grain length, and grain length/width ratio were observed between ZG78 and ZG99 (Figure 2). Only the seed setting rate and 1000-grain weight were significantly higher in ZG78 than those of ZG99. The seed setting rate was 93.4% and 90.3% in ZG78 and ZG99 (Figure 2f), respectively, and the 1000-grain weight was 26.8 g and 25.1 g (Figure 2g). It was interesting to discover whether ZG78 and ZG99 with similar backgrounds have differential responses to various stresses. Thus, ZG78 and ZG99 were further used to detect the seedling growth under 150 mM NaCl, 75 mM Na_2_SO_4_, 75 mM MgCl_2_ and 150 mM Mannitol for 7 days (Figure 3a,b). The seedling growth of ZG78 and ZG99 was more inhibited by NaCl and Na_2_SO_4_ treatments than that of MgCl_2_ and mannitol treatments. For example, NaCl inhibited the survival percentage by approximately 100% and seedling fresh weight by approximately 65% compared with the values obtained for the H_2_O condition in ZG99, while the corresponding values for mannitol treatment were approximately 10% and 5% (Figure 3c,d). Similarly, NaCl inhibited the survival percentage by approximately 87.5% and seedling fresh weight by approximately 60% compared with the values obtained for the H_2_O condition in ZG78, while mannitol did not inhibit both traits. This suggests that the seedling growth was more affected by Na^+^ than that of Cl^−^ and osmotic stress in both cultivars, and ZG78 had better stress tolerance compared with ZG99. 

### 2.3. Differential Expression of Na^+^/K^+^ Homeostasis-Associated Genes

**Figure 4:**
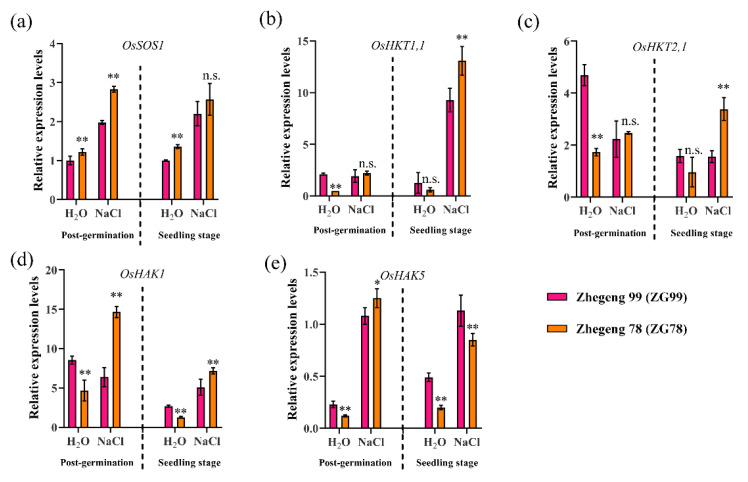
Differential expression of Na^+^/K^+^ homeostasis-associated genes between salt-tolerant Zhegeng 78 and salt-sensitive Zhegeng 99 cultivars determined by quantitative RT-PCR. The expression of (**a**) *OsSOS1*, (**b**) *OsHKT1,1*, (**c**) *OsHKT2,1*, (**d**) *OsHAK1*, and (**e**) *OsHAK5* in the post-germinated seeds and in three-leaf seedlings under the control (H_2_O) and post-150-mM-NaCl conditions for 12 h was normalized to that of *OsActin* gene control. Expression is relative to that in the *OsSOS1* of Zhenggeng 99 under H_2_O conditions, the value of which was set as 1. Data are means (±SD), *n* = 3. Significant differences between cultivars were determined using Student’s *t*-test: * *p* < 0.05; ** *p* < 0.01; n.s. not significant.

Maintaining Na^+^/K^+^ homeostasis is important for salt tolerance in rice. To expand our understanding of the differential salt responses in the salt-tolerant Zhegeng 78 (ZG78) and salt-sensitive Zhegeng 99 (ZG99) cultivars, which have similar genetic backgrounds, we determined the expression of genes associated with Na^+^/K^+^ homeostasis at the post-germination and seedling stages. Quantitative RT-PCR analysis demonstrated that the expressions of *OsSOS1*, *OsHAK1*, and *OsHAK5* were significantly induced by 150 mM NaCl stress for 12 h in the salt-tolerant ZG78 at the post-germination stage, while only *OsSOS1* and *OsHAK5* were significantly induced in the salt-sensitive ZG99 (Figure 4). The expression of *OsSOS1*, *OsHAK1*, and *OsHAK5* was approximately 2.4, 3.1, and 10.4 folds higher, respectively, under salt stress compared with those obtained for the H_2_O condition in ZG78 (Figure 4a,d,e), while the corresponding values for *OsSOS1* and *OsHAK5* were approximately 2.0 and 4.7 fold higher, respectively, in ZG99 (Figure 4a,e). Meanwhile, the expressions of *OsSOS1*, *OsHKT1,1*, *OsHTK2,1*, *OsHAK1*, and *OsHAK5* were significantly induced by 150 mM NaCl stress for 12 h in the salt-tolerant ZG78 at the seedling stage, while *OsSOS1*, *OsHKT1,1 OsHAK1*, and *OsHAK5* were significantly induced in the salt-sensitive ZG99. The expression of *OsSOS1*, *OsHKT1,1*, *OsHTK2,1*, *OsHAK1*, and *OsHAK5* was approximately 1.9, 22.0, 3.5, 5.6, and 4.25 folds higher, respectively, under salt stress compared with those obtained for the H_2_O condition in ZG78 (Figure 4a–e), while the corresponding values for *OsSOS1*, *OsHKT1,1 OsHAK1*, and *OsHAK5* were approximately 2.2, 7.3, 1.9, and 2.3 folds higher, respectively, in ZG99 (Figure 4a,b,d,e). Overall, significantly higher expressions of *OsSOS1*, *OsHAK1*, and *OsHAK5* at the post-germination stage and higher expressions of *OsHKT1,1*, *OsHTK2,1*, and *OsHAK1* at the seedling stage were observed in the salt-tolerant ZG78 compared with the corresponding values obtained for the salt-sensitive ZG99. These results indicate that the significantly higher induction of genes associated with Na^+^/K^+^ homeostasis in the salt-tolerant ZG78 might contribute to salt tolerance at the post-germination and seedling stages. 

### 2.4. Differential Accumulation of Na^+^ and K^+^

**Figure 5:**
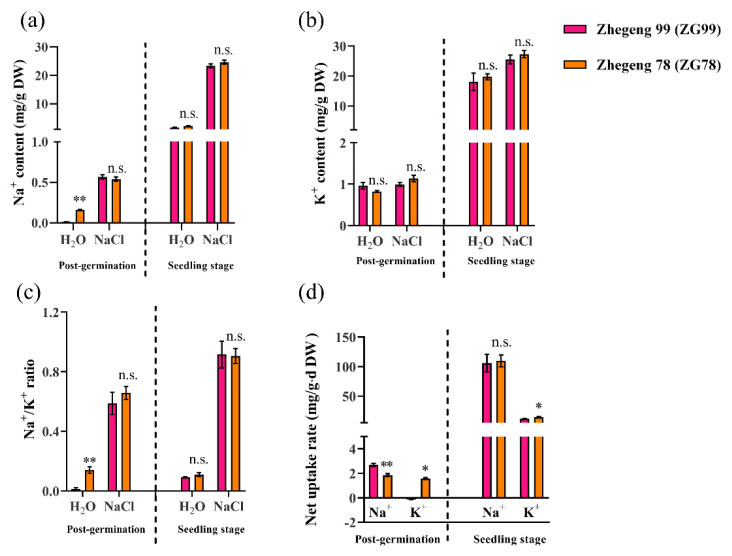
Differential accumulation of Na^+^ and K^+^ between salt-tolerant Zhegeng 78 and salt-sensitive Zhegeng 99 *japonica* rice cultivar. The (**a**) Na^+^ content, (**b**) K^+^ content, (**c**) Na^+^ /K^+^ ratio, and (**d**) Na^+^ and K^+^ net uptake rate in the post-germinated seeds and in three-leaf seedlings after 150 mM NaCl exposure for 24 h and 72 h at the post-germination and seedling stage, respectively. The H_2_O condition was used as control. Data are means (±SD), *n* = 3. Significant differences between cultivars were determined using Student’s *t*-test: * *p* < 0.05; ** *p* < 0.01; n.s. not significant.

To further determine the reasons for the differential salt tolerance between Zhegeng 78 (ZG78) and Zhegeng 99 (ZG99), the Na^+^ and K^+^ content, Na^+^ and K^+^ net uptake rate, and the Na^+^/K^+^ ratios were compared between ZG78 and ZG99 at the post-germination and seedling stages. The Na^+^ content was significantly increased by 150 mM NaCl treatment compared with that of H_2_O condition in both cultivars at the post-germination and seedling stages (Figure 5a). The Na^+^ content was approximately 3.38 and 11.33 folds higher at the post-germination and seedling stage, respectively, under salt stress compared with the equivalent values obtained under the H_2_O condition in ZG78, while the corresponding values were approximately 58 and 17 folds higher, respectively, in ZG99. However, the significant increase in K^+^ content caused by 150 mM NaCl treatment was only observed at the seedling stage in both cultivars (Figure 5b). The K^+^ content was approximately 1.39 and 1.38 folds higher in ZG78 and ZG99, respectively, under salt stress compared with the equivalent values obtained for the H_2_O condition. Although a higher Na^+^ content and Na^+^/K^+^ ratio was observed in ZG78 compared with ZG99 at the post-germination stage under the H_2_O condition, no significant differences in Na^+^ and K^+^ contents, as well as in the Na^+^/K^+^ ratio, were observed between ZG78 and ZG99 at both the post-germination and seedling stages under salt stress (Figure 5a–c). However, a significantly lower Na^+^ net uptake rate at the post-germination stage and a higher K^+^ net uptake rate at the post-germination and seedling stages were observed in ZG78 compared with those of ZG99 under salt stress (Figure 5d). The Na^+^ net uptake rate was 1.85 and 2.69 mg/g·d dry weight (DW) in ZG78 and ZG99, respectively, at the post-germination stage under salt stress. The K^+^ net uptake rates were 1.57 and −0.13 mg/g·d DW in ZG78 and ZG99 at the post-germination stage, respectively, while the corresponding values were 14.49 and 11.89 mg/g·d DW at the seedling stage under salt stress. These results indicate that a significantly lower Na^+^ net uptake rate and a higher K^+^ net uptake rate in ZG78 might be contributing to its salt tolerance. 

### 2.5. Differential Accumulation of Hydrogen Peroxide

**Figure 6:**
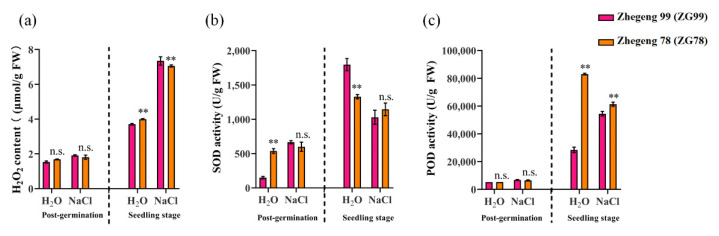
Differential H_2_O_2_ accumulation and SOD and POD activity between salt-tolerant Zhegeng 78 and salt-sensitive Zhegeng 99 *japonica* rice cultivar. The (**a**) H_2_O_2_ content, (**b**) SOD activity, and (**c**) POD activity in the post-germinated seeds and in three-leaf seedlings after 150 mM NaCl exposure for 24 h and 72 h at the post-germination and seedling stage, respectively. The H_2_O condition was used as control. Data are means (±SD), *n* = 3. Significant differences between cultivars were determined using Student’s *t*-test: ** *p* < 0.01; n.s. not significant.

The production of ROS such as hydrogen peroxide (H_2_O_2_) is known to be increased under stress conditions. To determine whether differential H_2_O_2_ accumulation caused the differential salt tolerance between Zhegeng 78 (ZG78) and Zhegeng 99 (ZG99), the levels of H_2_O_2_ were determined and compared between the salt-tolerant ZG78 and the salt-sensitive ZG99 at the post-germination and seedling stages. The levels of H_2_O_2_ were significantly lower in ZG 78 than the corresponding values obtained for ZG99 after 150 mM NaCl treatment for 72 h only at the seedling stage (Figure 6a). The H_2_O_2_ content was 7.05 and 7.34 μmol/g fresh weight (FW) in ZG78 and ZG99, respectively, at the seedling stage under salt stress. Antioxidant enzymes, including SOD and POD, are involved in the defenses against ROS. The significant difference of SOD activity between ZG78 and ZG99 was only observed under the H_2_O condition (Figure 6b). Significantly higher SOD activity was observed in ZG78 compared to ZG99 at the post-germination stage under the H_2_O condition, while significantly lower SOD activity was observed in ZG78 at the seedling stage. However, the significant difference of POD activity between ZG78 and ZG99 was only observed at the seedling stage (Figure 6c). Significantly higher POD activity was observed in ZG78 compared to ZG99 at the seedling stage under both H_2_O and salt stress conditions (Figure 6c). The POD activity was 83,029.42 and 28,422.22 U/g FW in ZG78 and ZG99 under H_2_O conditions, respectively, while the corresponding values were 61,343.28 and 54,498.98 U/g FW under salt stress. These results suggest that differences in the accumulation levels of H_2_O_2_ might cause the differential salt responses between ZG78 and ZG99 at the seedling stage.

## 3. Discussion

Good performance in terms of seedling establishment and seedling growth is an essential characteristic for direct seeding in rice. Previous studies on salt tolerance were mainly focused on seed germination and seedling stages in rice [2,4,13]. Usually, the sprouted rice seeds are broadcast on puddled soils for direct seeding [23]. Thus, the evaluation of salt tolerance was conducted at both the post-germination (root length 5 mm) and seedling (three-leaf) stages in this study. Previously, several salt-tolerant rice species, such as wild rice accessions [19,20], Chinese landraces Sea Rice 86 [21], Changmaogu [22], and Jiucaiqing [13], have been used to investigate salt tolerance. However, these salt-tolerant accessions could not be popularly cultivated due to their unfavorable agronomic traits. Therefore, we used the six currently cultivated *japonica* rice varieties to evaluate the salt tolerance for future direct seeding in this study. Typically, 100 mM to 150 mM of NaCl solution was used to evaluate salt tolerance in rice in previous studies [2,4], and thus, 150 mM NaCl solution was used in this study. It was reported that there was significant natural variation in seed germination and seedling growth among *indica* accessions under salt stress [2]. Similarly, there was significant variation of seedling growth under salt stress at both the post-germination and seedling stages in six *japonica* rice cultivars in this study. We observed that 150 mM NaCl treatment significantly inhibited root growth and seedling survival in *japonica* rice cultivars. ZG78 and XS134 simultaneously exhibited relatively higher salt tolerance at both the post-germination and seedling stages, while ZG99 and ZHG25 had lower salt tolerance. Interestingly, ZG78 and ZG99 have similar genetic backgrounds but they showed differential salt responses at the post-germination and seedling stages. Therefore, ZG78 and ZG99 were used to further investigate the differential physiological responses to salt stress between salt-sensitive and salt-tolerant *japonica* rice cultivars. 

Salt stress is usually associated with an excessive amount of NaCl causing ion toxicity. Several key genes associated with Na^+^/K^+^ homeostasis have been reported in rice. The *OsSOS1* gene regulates the net root Na^+^ uptake [5], *OsHAK1* [10] and *OsHAK5* [11] activate K^+^ uptake in rice, and the sodium transporter genes *OsHKT1;1*, *OsHKT2;1*, and *OsHAK1* are involved in the K^+^ uptake [6,7,8]. Thus, the expressions of five genes including *OsSOS1*, *OsHKT1,1*, *OsHTK2,1*, *OsHAK1*, and *OsHAK5* were compared between salt-tolerant ZG78 and salt-sensitive ZG99 at both the post-germination and seedling stages to reveal their differential physiological responses to salt stress in this study. We observed that the expression of *OsHAK1* was specifically induced by salt stress at the post-germination stage in ZG78 compared with that of the H_2_O condition, and the expression of *OsHTK2,1* was specifically induced at the seedling stage. However, these two genes were not induced by salt stress in ZG99. We assumed that the differential expressions of *OsHAK1* and *OsHTK2,1* under salt stress might be contributing factors to the differential salt tolerance between ZG78 and ZG99. A previous study showed that the salt tolerance of rice at the seed germination and seedling stages might be regulated by differential genetic factors [2]. Similarly, we observed that ZG78 had significantly higher expressions of *OsSOS1*, *OsHAK1*, and *OsHAK5* compared with those of salt-sensitive ZG99 at the post-germination stage, while ZG78 had higher expressions of *OsHKT1,1 OsHKT2,1*, and *OsHAK1* at the seedling stage. It is suggested that the higher salt tolerance of ZG78 at the post-germination and seedling stages might be regulated by differential genes. The above-mentioned genes are associated with Na^+^/K^+^ homeostasis in seedlings of rice [5,6,7,8,10,11]. Thus, the Na^+^ and K^+^ contents, Na^+^/K^+^ ratio, and Na^+^ and K^+^ net uptake rates were further compared between ZG78 and ZG99 at the post-germination and seedling stages in this study. Under salt stress, lower Na^+^ and higher K^+^ net uptake rates were only observed in ZG78 as compared to ZG99, while no significant differences in terms of Na^+^ and K^+^ contents and Na^+^/K^+^ ratios were found between ZG78 and ZG99. Overall, the K^+^ uptake is regulated by several genes such as *OsHKT1;1* [6], *OsHKT2;1* [8], *OsHAK1* [10], and *OsHAK5* [11] in rice; however, the differential contributions of each gene to K^+^ uptake between ZG78 and ZG99 need further investigation in the future.

ROS such as H_2_O_2_ play a dual role in seed germination as messengers at low concentrations and as toxic products at high concentrations [14]. The excessive accumulation of ROS disturbs ionic homeostasis in the cells under stress [24]. Thus, the relationship between ROS production and salt tolerance was analyzed to determine whether differential H_2_O_2_ accumulation caused the difference in salt tolerance between salt-tolerant ZG78 and salt-sensitive ZG99 at both the post-germination and seedling stages in this study. We firstly observed that ZG78 had a significantly lower level of H_2_O_2_ than that of ZG99 only at the seedling stage while not the post-germination stage under salt stress. This suggested that the differential H_2_O_2_ level might be partly explained by the differential salt tolerance between ZG78 and ZG99, especially at the seedling stage. To cope with stress, plants have evolved a wide range of antioxidant systems, such as the antioxidant enzymes SOD and POD, to scavenge excessive ROS. Superoxide radicals produced in the plant cells are firstly dismutated to H_2_O_2_ by the SOD action [25]. Then, POD catalyzes the H_2_O_2_ oxidoreduction by transferring electrons from various donor molecules [26,27,28]. In order to further explain the differential H_2_O_2_ accumulation between ZG78 and ZG99, the comparison of SOD and POD activities was conducted between ZG78 and ZG99 at both the post-germination and seedling stages. Our results showed that there were no significant differences in SOD and POD activities between ZG78 and ZG99 under salt stress at the post-germination stage. However, we observed that the POD activity was significantly higher in ZG78 than in ZG99 under salt stress at the seedling stage, while no significant difference in SOD activity was observed at the post-germination stage. It is suggested that the lower H_2_O_2_ accumulation in the salt-tolerant ZG78 was possibly caused by POD action at the seedling stage. In this situation, the differential H_2_O_2_ level might be partly explained the differential salt tolerance between ZG78 and ZG99. 

## 4. Materials and Methods

### 4.1. Plant Materials and Growth Conditions

Six currently cultivated *japonica* rice varieties, including Zhegeng 78 (ZG78), Zhegeng 88 (ZG88), Zhegeng 99 (ZG99), Zhegeng 100 (ZG100), Zhehugeng 25 (ZHG88), and Xiushui 134 (XS134), from Zhejiang province (E 118°01′–123°10′, N 27°02′–31°11′) of China were used in this study. The plants were grown in the experimental fields in Zhejiang Academy of Agricultural Sciences according to the local cultivation conditions (Hangzhou, Zhejiang, China). The average temperature, annual precipitation, annual precipitation days, and annual sunshine hours are approximately 18.4 °C, 1378.5 mm, 134 days, and 1471.2 h in Hangzhou, respectively. The plants of each cultivar were planted in plots of 10 square meters with randomized block designs. Three replicates were used. The mature seeds were harvested and dried at 42 °C for 7 days to release seed dormancy [4], and then well-filled seeds were randomly selected for the experiments.

### 4.2. Evaluation of Stress Tolerance

The evaluation of stress tolerance consisted of two independent experiments. Firstly, the above-mentioned cultivars ZG78, ZG88, ZG99, ZG100, ZHG88, and XS134 were used to select the salt-tolerant and salt-sensitive cultivars at the post-germination and seedling stages. Typically, 100 mM to 150 mM NaCl solution was used to evaluate salt tolerance in rice [2,4], and thus 150 mM NaCl solution was used in this study. Thirty post-germinated seeds (radicle length 5 mm) per replicate of each cultivar were sowed in 96-well plates and added to 150 mM NaCl for 7 days at 25 °C. After the treatments, the lengths of the shoots and roots were compared to evaluate salt tolerance among cultivars at the post-germination stage. Meanwhile, 30 seeds per replicate of each cultivar were sowed in 96-well plates and seedling growth continued up to the three-leaf stage during 15 days of cultivation at 25 °C in nutrient solution (Beijing Kulaibo Technology Co., Ltd., Beijing, China). The effective components of nutrient solution mainly included NH_4_NO_3_, NaH_2_PO_4_·2H_2_O, K_2_SO_4_, CaCl_2_, MgCl_2_, and (NH_4_)_2_SO_4_. After that, the seedlings were treated under 150 mM NaCl condition for 7 days and recovery growth for 7 days at 25 °C. After treatments, the seedling survival was compared to evaluate salt tolerance among cultivars at the seedling stage.

Secondly, the salt-tolerant and salt-sensitive cultivars were selected according to the first experiment for further confirmation of their differential responses to various stresses. Evaluation of the salt stress indicated that ZG78 and ZG99 were salt-tolerant and salt-sensitive cultivars, respectively, at both the post-germination and seedling stages. Interestingly, ZG78 and ZG99 have similar genetic grounds, and thus, we were interested in revealing the reasons for the differential responses to salt stress between ZG78 and ZG99 despite their genetic similarities. Thus, the confirmation of differential responses to various stresses was conducted using ZG78 and ZG99 at the seedling stage. Thirty seeds per replicate of ZG78 and ZG99 were sowed in 96-well plates and seedling growth continued up to the three-leaf stage during 15 days of cultivation at 25 °C in nutrient solution (Beijing Kulaibo Technology Co., Ltd., Beijing, China). After that, the seedlings were treated with 150 mM NaCl, 75 mM Na_2_SO_4_, 75 mM MgCl_2_ and 150 mM Mannitol for 7 days at 25 °C. After the treatments, the survival percentage and seedling fresh weight were measured. Three biological replicates were used for each experiment.

### 4.3. Evaluation of Agronomic Traits

The plants of ZG78 and ZG99 were grown in the experimental fields in Zhejiang Academy of Agricultural Sciences according to the local cultivation conditions (Hangzhou, Zhejiang, China). The plants of each cultivar were planted in 10 square meter plots with randomized block designs, as mentioned above. Three replicates were used. Seven agronomic traits including whole growth period, plant height, total grain number per panicle, filled grain number per panicle, seed setting rate, 1000-grain weight, grain yield, brown rice rate, milled rice rate, grain length, and grain length/width ratio were determined.

### 4.4. Quantitative Reverse Transcription PCR

Total RNA extraction and first-strand cDNA synthesis were conducted using an HP Plant RNA Kit (Omega, Atlanta, GA, USA) and HiScript Reverse Transcriptase (Vazyme, Nanjing, China), respectively, according to the manufacturer’s instructions. Quantitative reverse transcription PCR (qRT-PCR) was carried out according to Zhao et al. [29] using a CFX96 Real-Time System (Bio-Rad, Hercules, CA, USA). The PCR conditions were as follows: 95 °C for 2 min, followed by 40 cycles of 95 °C for 5 s, and 60 °C for 10 s. The primers are listed in Supplementary Table S1. The rice *OsActin* gene was used as an internal control. The comparative *C*_T_ method was used to calculate the transcript levels [30]. Three biological replicates were used.

### 4.5. Determination Evaluation of K^+^ and Na^+^ Contents

The determination of K^+^ and Na^+^ contents was carried out according to He et al. [13]. The plants of each sample were dried at 95 °C for 30 min and then 55 °C for 7 days. After that, each sample was grinded into powder and 0.1 g dry weight (DW) powder was extracted. The contents of K^+^ and Na^+^ were detected using inductively coupled plasma mass spectrometry (ICP-MS; Perkin Elmer, Waltham, MA, USA). The K^+^ or Na^+^ contents and their net uptake rates were expressed as mg/g DW and mg/g DW d^−1^, respectively. Three biological replicates were used.

### 4.6. Determination of Hydrogen Peroxide (H_2_O_2_) Level and Enzyme Activities

Each harvested fresh sample was rapidly frozen in 1 mL of cold acetone (4 °C) and homogenized into a powder. Then, the H_2_O_2_ levels and the activities of SOD and POD were assessed using the commercial assay kits according to the manufacturer’s instructions (Suzhou Keming Bioengineering Company, Suzhou, Jiangsu, China). The absorbance was determined at 415 nm, 560 nm, and 470 nm for H_2_O_2_, SOD, and POD, respectively, using the supernatant extracted from 0.1 g fresh weight (FW) of sample. One unit (U) of SOD and POD activity was defined as the amount required to inhibit the photoreduction of nitroblue tetrazolium (NBT) by 50% and to cause an absorbance change of 0.005 units per minute, respectively. The H_2_O_2_ content and SOD and POD activity were expressed as μmol/g FW and U/g FW, respectively. Three biological replicates were used.

### 4.7. Data Analysis

GraphPad Prism software (version 9.0) was used for data analysis, and the Student’s *t*-test was used to detect the significant differences between samples.

## 5. Conclusions

In conclusion, the currently cultivated *japonica* rice cultivars were used to evaluate salt tolerance in this study. We observed that there was natural variation in salt tolerance in *japonica* rice cultivars. ZG78 and XS134 had better salt tolerance simultaneously at both the post-germination and seedling stages, while ZG99 and ZHG25 had lower salt tolerance. One salt-tolerant ZG78 and one salt-sensitive ZG99, with similar genetic backgrounds, were thus used to reveal the differential responses to salt stress at the post-germination and seedling stages. The higher expression of Na^+^/K^+^ homeostasis-related genes, such as *OsSOS1*, *OsHAK1*, and *OsHAK5*, at the post-germination stage, and of *OsHKT1,1*, *OsHTK2,1*, and *OsHAK1* at the seedling stage, in ZG78, might have caused the higher K^+^ net uptake rates that contributed to salt tolerance. Meanwhile, the higher POD activity at the seedling stage might be associated with the decline in H_2_O_2_ accumulation, which contributed to the salt tolerance in ZG78. The differential expression of other Na^+^/K^+^ homeostasis-related genes in salt-tolerant ZG78, and the activities of other ROS scavenging enzymes and antioxidants, need to be further investigated. The salt-tolerant ZG78 with favorable agronomic traits might be valuable in the future cultivation of salinity soils.

## Data Availability

Not applicable.

## Supplementary Materials

The following are available online at https://www.mdpi.com/article/10.3390/plants10112433/s1, Table S1. List of primer pairs used in this study.
