# Supplementary material for: Differential Physiological Responses to Salt Stress between Salt-Sensitive and Salt-Tolerant japonica Rice Cultivars at the Post-Germination and Seedling Stages
Source: Plants (Basel). 2021 Nov 11;10(11):2433. doi: 10.3390/plants10112433 (PMC8696616; doi:10.3390/plants10112433)
Supplement: Supplementary file 1 [file plants-10-02433-s001.zip › Supplemental Table 1.pdf]

Supplemental Table S1. The primer pairs used in this study.

Primer name	Sequence (5'–3')
OsSOS1-F	CTCCGTGCTCATAGAATCGC
OsSOS1-R	ATACTCACTCAAGTGGGTCAATACC
OsHAK1-F	GTTGATGATGCTGATGTTGGAAG
OsHAK1-R	CCAACACTTTCAGCTGAAAC
OsHAK5-F	CTTGGAAATCTGAGTAAGTACTC
OsHAK5-R	CGAATCTCCATGCATGTTCTG
OsHKT1,1-F	TTCACCACTCTTGCGGCTATG
OsHKT1,1-R	TGTTTGTAGCCAGTCTCCCCAG
OsHKT2,1-F	CACAGTCTCCTCGTTTGCGAA
OsHKT2,1-R	GCAAGAATCTGGCCGATGAA
OsActin-F	AGGAAGGCTGGAAGAGGACC
OsActin-R	CGGGAAATTGTGAGGGACAT
